# How parental factors shape the plant embryo

**DOI:** 10.1042/BST20240369

**Published:** 2025-01-21

**Authors:** Alexa-Maria Wangler, Martin Bayer

**Affiliations:** Centre for Plant Molecular Biology, University of Tübingen, Tübingen 72076, Germany

**Keywords:** *Arabidopsis thaliana*, embryogenesis, parental conflict, suspensor, zygote polarization

## Abstract

Primary axis formation is the first step of embryonic patterning in flowering plants and recent findings highlight the importance of parent-of-origin effects in this process. Apical-basal patterning has a strong influence on suspensor development, an extra-embryonic organ involved in nutrient transport to the embryo at an early stage of seed development. The endosperm, a second fertilization product, nourishes the embryo at later stages of seed development. Parent-of-origin effects are phenotypic effects that depend on whether a causal gene is inherited from the mother or the father. They are discussed in the context of the parental conflict theory in relation to nutrient allocation to the offspring. Imprinting is an important mechanism leading to uniparental gene expression in the endosperm and maternal control of its development. The parental conflict theory would predict that, with limited resources available, there is a competition between paternal alleles to increase nutrient supply, allowing rapid development and seed filling. A parental conflict might therefore shape the evolution of genes that can influence the allocation of nutrients to the seeds. However, we will also discuss other possible causes that might select genes for uniparental contribution. New data show that parent-of-origin effects also occur during the early stages of embryo development. These appear to be caused primarily by the carry-over of gamete-derived factors. In this review, we will highlight the molecular pathways that control apical-basal patterning in the early embryo and discuss recent findings in the context of the parental conflict theory and alternative explanations.

## Introduction

In flowering plants, seeds serve as a multigenerational home. The developing embryo is encapsulated by layers of maternal seed tissue while being nourished by its altruistic sibling, the endosperm [1]. Precise growth coordination between the seed coat and the two fertilization products—embryo and endosperm—is therefore essential for successful seed development [2]. This process presumably requires intricate communication between sporophytic and gametophytic tissues during early ovule development prior to fertilization. After fertilization, there is an even greater need for communication between the maternal tissues (seed coat and nucellus) and the fertilization products (embryo and endosperm) in the rapidly growing seed. Because of this growth co-ordination by the maternal seed coat, one would expect strong maternal effects on embryo and endosperm development. Paternal effects would not be expected in this context. However, they might arise from other necessities, such as competition between paternal alleles and parental conflict (discussed below).

Parent-of-origin effects are phenotypic effects of genes that depend on whether the gene is inherited from the mother or the father. Several studies have reported such effects during seed development, mainly in the endosperm [3]. This is most likely due to the unique genomic composition of this tissue. During the development of the precursor of the endosperm, two haploid polar nuclei fuse to form the homodiploid central cell nucleus. After fertilization, the endosperm is therefore triploid and contains twice as much maternal genome as the paternal genome [1]. To prevent genomic imbalance, this can lead to preferential expression of genes from one parent. There are excellent reviews available on parent-of-origin effects and imprinting in the endosperm; therefore, this will not be the focus of this review [4–6]. Here, we will focus specifically on parent-of-origin effects in the early embryo and discuss recent findings in the model species *Arabidopsis thaliana*.

### Early events in *Arabidopsis* embryogenesis

**Figure 1:**
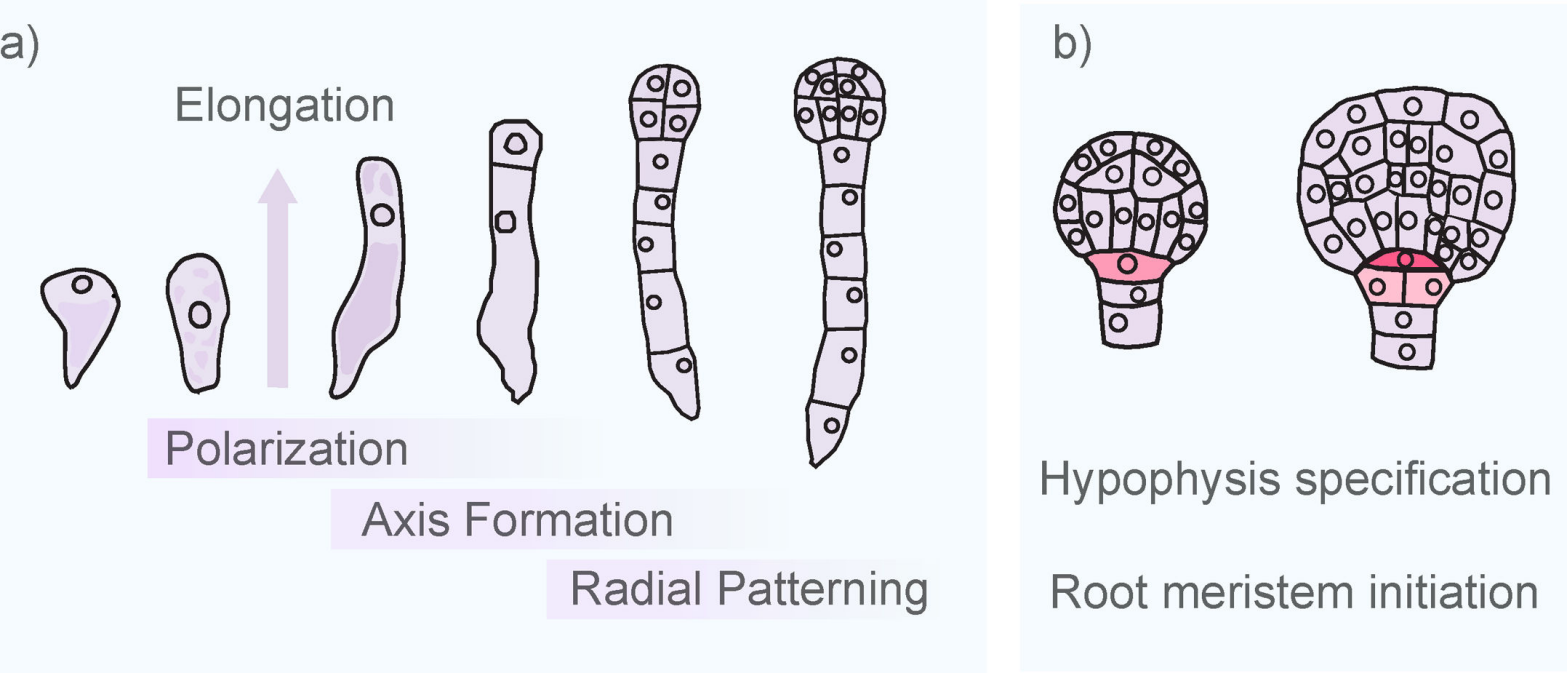
*Arabidopsis* embryogenesis. (**a**) Zygote polarization initiates the patterning process with an asymmetric cell division. The smaller apical cell forms the embryo proper while the larger basal cell gives rise to the mostly extraembryonic, filamentous suspensor. (**b**) The uppermost cell of the suspensor (hypophysis, red) divides asymmetrically to form a lens-shaped cell (dark red), the precursor of the organizing center of the root stem cell niche, and the columella precursor cells (light red).

The *A. thaliana* egg cell is a polar structure, with its nucleus at the apex and a large vacuole at the base. After fertilization, however, polarity is apparently lost, accompanied by repositioning of the nucleus toward the cell center and fragmentation of the vacuole [7] (Figure 1a). After a short period, the zygote begins to repolarize while elongating approximately threefold, the nucleus migrates to the apical region, and a large vacuole reforms toward the basal end [7]. This repolarization of the zygote anticipates the first asymmetric division which results in two daughter cells with different developmental fates. A smaller apical cell that develops into the embryo proper, and a larger basal cell that gives rise to the extraembryonic suspensor [8].

Two rounds of longitudinal divisions and one transverse division in the apical cell form a spherical eight-cell pro-embryo. Each of these cells then undergoes a tangential division, defining an outer and inner layer at the 16-cell stage (Figure 1a). A series of asymmetric cell divisions establishes the stem cell niches of the later shoot and root meristems. While the rest of the suspensor remains extraembryonic, the uppermost descendant of the basal cell, called the hypophysis, is in contact with the embryo and is recruited to embryonic development. It divides asymmetrically to form a smaller lens-shaped cell, the precursor of the organizing center of the root stem cell niche, and a larger cell, the precursor of the root cap [9,10] (Figure 1b).

Local cell divisions in the embryo proper lead to a shift in embryo symmetry from radial (at the globular stage) to bilateral, known as the heart stage. At this stage, the precursor cells, that will give rise to the major seedling organs, are already recognizable [11]. As the embryo continues to grow, it will eventually occupy the entire lumen of the seed, taking nutrients from the degenerating endosperm [12,13]. Before the initiation of programmed cell death in the endosperm, however, the suspensor is an important conduit for nutrients and plant hormones [14,15]. Furthermore, it pushes the embryo from the micropylar region into a more central region of the endosperm. This might facilitate the subsequent uptake of endosperm-derived nutrients by the embryo [16]. The observation that shorter suspensor length correlates with slower embryo growth highlights this important role [17]. Interestingly, several genes involved in the suspensor development show parent-of-origin effects [17–22]. This is quite exceptional as most developmental processes in the early embryo are controlled by transcripts that are contributed equally by both parental alleles [23,24]. Consequently, most embryonic mutations are zygotic recessive [25]. This is not surprising when observing histone dynamics during and after fertilization, as gametic histones appear to be rapidly turned over and replaced by zygotic histones [26,27]. Consequently, imprinting of parental alleles and uniparental expression during later stages of embryogenesis would require a specialized mechanism. In contrast, during the early events of embryogenesis, the carry-over of parental factors from the gametes can be a major source of parent-of-origin effects independent of imprinting. The extent of maternal effects on the earliest events of embryogenesis is a matter of debate [24,28–32].

## Introducing the molecular players

**Figure 2:**
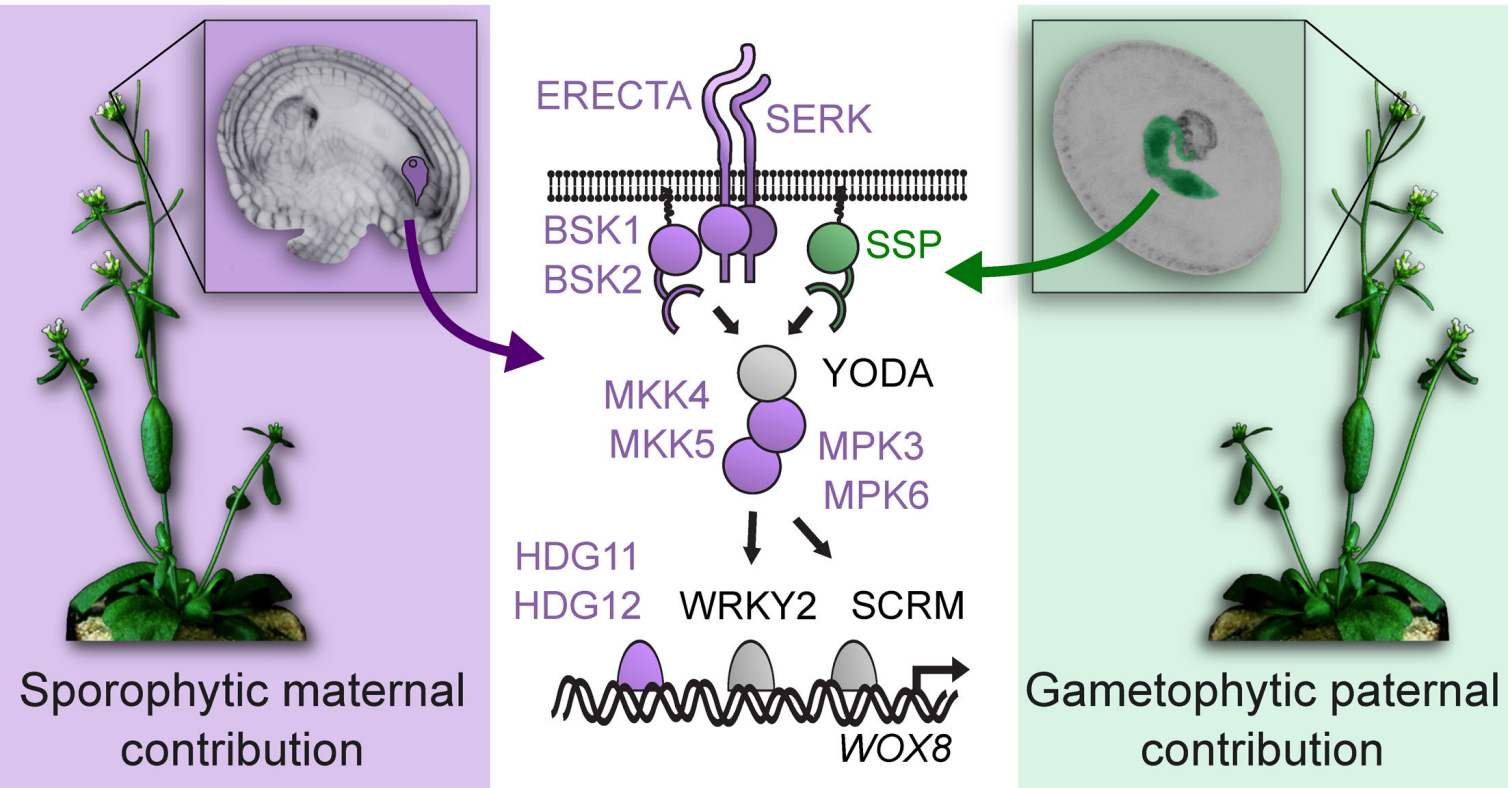
Parental contributions to the ERECTA-YODA MAP kinase signaling pathway. The molecular architecture of the embryonic ERECTA-YODA signaling pathway is shown in a simplified, schematic depiction in the center. Female and male contributions are shown in purple and green, respectively. Zygotic factors are shown in gray. A receptor kinase complex, consisting of ERECTA- and SOMATIC EMBRYOGENESIS RECEPTOR-LIKE KINASE (SERK) family receptor kinases, activates the YODA (YDA)-dependent MAP kinase cascade via the membrane-associated pseudokinases BSK1 and BSK2. The expression of the homeodomain transcription factor gene *WOX8* depends on the presence of HDG11 and HDG12 and on MAP kinase-mediated phosphorylation of the transcription factors WRKY2 and SCREAM/ICE1 (SCRM). Large parts of this pathway are contributed by the maternal sporophyte before fertilization (purple). The MAPKK kinase YODA can be activated in a receptor-independent manner by the BRASSINOSTEROID SIGNALING KINASE (BSK) family protein SSP. SSP has evolved as a naturally occurring, constitutively active BSK variant and is delivered by the male gamete (sperm cell, green) during fertilization.

The first step of the embryonic patterning process in *A. thaliana* is the polarization and asymmetric cell division of the zygote [33]. This break in symmetry initiates differential gene expression and the acquisition of different cell identities in the daughter cells and starts the patterning process of the embryo [8]. At the molecular level, this is accomplished at least in part by a mitogen-activated protein (MAP) kinase signaling pathway (Figure 2).

This receptor kinase/MAP kinase signaling pathway is involved in many cell fate decisions during plant development [18,33–41]. In different developmental contexts, it universally consists of a receptor kinase complex in the plasma membrane that includes receptor kinases of the ERECTA family (ERf) and the SOMATIC EMBRYOGENESIS RECEPTOR-LIKE KINASE (SERK) co-receptor family [35,42,43]. They perceive small extra-cellular, cysteine-rich proteins of the EPIDERMAL PATTERNING FACTOR-LIKE (EPFL) family [38,44]. Two membrane-associated pseudokinases of the BRASSINOSTEROID SIGNALING KINASE (BSK) family, BSK1 and BSK2, link receptor signaling to MAP kinase activation by interacting with the MAPKK kinase YODA (YDA) [21,39,40]. The MAP kinase cascade furthermore includes MAP KINASE KINASE 4 (MKK4) and MKK5 as well as the MAP kinases MAP KINASE 3 (MPK3) and MPK6 [20,45]. We will refer to this core pathway as the ERECTA-YODA pathway in this review. The downstream targets of this MAP kinase cascade seem to be context-dependent and quite diverse, including transcription factors of the basic helix-loop-helix (bHLH) and WRKY family transcription factors. In the embryo, MPK6-dependent phosphorylation of the bHLH factor SCREAM/ICE1 (SCRM) and the WRKY transcription factor WRKY2 activate the WUSCHEL-RELATED HOMEOBOX (WOX) transcription factor gene *WOX8* [46,47]. *WOX8* activation in the zygote also depends on the activity of HOMEODOMAIN GLABROUS11 (HDG11) and HDG12 [19].

In the context of zygote polarization, there is an intriguing deviation from the universal architecture of the ERECTA-YODA pathway. It is proposed that two independent inputs activate the YDA-dependent MAP kinase cascade that are contributed by the female and male gametes, respectively [22] (Figure 2). The receptor kinase ER appears to be contributed exclusively by the maternal plant as *er* mutants exhibit a sporophytic maternal effect. This can be demonstrated in reciprocal crosses between *er* single or *er erl* double mutants and wild-type plants, respectively. Only if the mutant allele is contributed by the maternal crossing partner, the embryo will show defects in zygote polarization and embryonic patterning, regardless of the genotype of the paternal crossing partner. Furthermore, maternal plants must be homozygous for the *er* mutation to cause defective phenotypes in the embryo, indicating that the maternal effect is not gametophytic (depending on the genotype of the egg cell) but sporophytic (depending on the genotype of the diploid maternal plant). The reason for this unusual parental effect is that the *ER* gene products are already produced pre-meiotically in the megaspore mother cell and are inherited through the female germline to the zygote [22].

A similar maternal effect can also be observed for *BSK1* and *BSK2*, *MKK4* and *MKK5*, *MPK3* and *MPK6*, and *HDG11* and *HDG12* [19,20,45]. This suggests that a large portion of this signaling pathway is solely produced by maternal alleles. In self-fertilizing, isogenic *A. thaliana* plants, this is of little consequence. In polymorphic populations, however, this would be relevant as only the genetic variation present in the diploid maternal plant would be reflected in the composition of these pathway components. For these proteins, the genotype of the pollen donor would be irrelevant.

This predominant maternal bias seems to be counterbalanced by a second, exclusively paternal input. The BSK family gene *SHORT SUSPENSOR* (*SSP* or *BSK12*) is a *Brassicaceae*-specific sister gene of *BSK1* that arose during the last whole-genome duplication at the origin of the *Brassicaceae* [18,21,48]. *BSK1* is broadly expressed and fulfills its canonical function as a signaling relay in many developmental contexts including early embryogenesis. In contrast, *SSP* shows a very narrow expression pattern. *SSP* transcripts accumulate specifically in sperm cells in mature pollen, whereas the protein only appears in the zygote after fertilization [18]. This tight control of expression at the transcriptional and post-transcriptional level is necessary because the SSP protein has lost regulatory residues and evolved as a naturally occurring, constitutively active BSK variant [21]. It has been shown that SSP can activate YDA in an ER-independent fashion [22]. This would argue that zygote polarization relies on two independent parental inputs: canonical receptor activation under maternal control and non-canonical activation of YDA by sperm cell-derived SSP (Figure 2). This raises the question of whether *SSP* has evolved under selective pressure to overcome the maternal control of ERECTA-YODA signaling. Alternatively, the selective advantage of such a two-component system could be in kickstarting the patterning process by mechanistically linking it to the fertilization event.

Recently, it has been shown that the precise control of transcription factor expression by gamete-derived factors is crucial for correct embryonic patterning and its failure can have long-lasting effects in the seedling and adult plant [49]. The sperm-derived C2H2 zinc finger domain repressors TRANSCRIPTIONAL REPRESSOR OF EIN3-DEPENDENT ETHYLENE-RESPONSE 1 (TREE1) and its homolog DUO-ACTIVATED ZINC FINGER3 (DAZ3) bind cis-regulatory elements in the promoter of the RWP-RK DOMAIN CONTAINING (RKD) transcription factor gene *RKD2. RKD2* is expressed in the egg cell but is rapidly downregulated after fertilization by the repressive activity of TREE1 and DAZ3. In the absence of TREE1 and DAZ3, ectopic expression of *RKD2* in the early embryo can in rare cases lead to misspecification of the hypophysis and incorrect patterning of the root stem cell niche. Parental effects in the early embryo can therefore have potentially long-lasting effects in the later plant.

Linking early embryonic patterning to the fertilization event by using gamete-derived two-component systems might therefore be a simple evolutionary solution for precise timing in early plant embryogenesis.

## The parental conflict theory

In sexually reproducing, outcrossing organisms where multiple offspring develop simultaneously, such as plants with multiple seeds in the same fruit, paternal effects on embryogenesis could lead to competition between paternal alleles. This might occur if they can influence the allocation of nutrients to the embryo. If they alter the source–sink relationship for nutrients between the maternal plant and the developing seeds in favor of the embryo carrying the paternal allele, an evolutionary arms race between them might arise. The aim would be to increase the supply of nutrients to the embryo and accelerate its growth in order to outcompete unrelated siblings. To prevent excessive use of maternal resources in such a scenario, there would be selective pressure to exert maternal control over processes that are targeted by competing paternal alleles. This evolution of parent-of-origin effects through competing maternal and paternal interests is predicted in the parental conflict theory [50].

The suspensor is thought to act as a conduit for nutrients and plant hormones during the early phase of embryogenesis [16]. The control of the extent of suspensor proliferation and its physiological role in promoting embryo growth and optimal embryonic positioning within the seed may therefore be at the center of a parental conflict. It is interesting to note that the known effects of gamete-derived parent-of-origin genes described in Arabidopsis which do not influence endosperm development, appear to mainly target suspensor development [17–22].

In this context, sporophytic maternal control of zygote polarization through premeiotic inheritance of ERECTA-YODA pathway components might be a simple way to ensure a uniform control of suspensor development in all seeds. The production of ERECTA-YODA components in the diploid generation prior to meiosis will bypass Mendelian segregation of maternal alleles and mask the individual effect of each allele. In contrast, the paternal gametophytic effect of *SSP* could be the result of competing paternal alleles aiming to overcome this maternal control to maximize resource allocation to their progeny.

In the hermaphrodite *A. thaliana*, a predominantly self-pollinating species, parental conflicts would not be expected. However, it should be emphasized that close relatives of *A. thaliana*, such as *A. lyrata,* are obligate outcrossing species. Given that *SSP* evolved as a sister gene of *BSK1* during the last whole-genome duplication at the advent of the *Brassicaceae* [48], the paternal control of *SSP* is expected in ancestral outcrossing species as well. The observed parental effects in *A. thaliana* could therefore be an evolutionary remnant of an existing parental conflict in outcrossing *Brassicaceae* species.

It will be fascinating to explore whether the parental control of the ERECTA-YODA pathway is the result of a parental conflict or an elegant way of synchronizing axis formation in the early embryo with the fertilization event. The acceleration of the embryonic patterning process may confer a selective advantage in opportunistic ruderal plant species. Further experiments are clearly needed to address these questions.

## Conclusion

In recent years, it has become clear that parental effects have a strong influence on the primary axis formation of the embryo and suspensor development in *A. thaliana*. These parental effects on apical-basal patterning are mainly caused by the carry-over of gamete-derived components of the ERECTA-YODA signaling pathway. The primary axis formation has a strong influence on suspensor development, a structure involved in nutrient supply during the early phases of seed development. It is therefore an open question whether the biparental control of suspensor development is the consequence of parental conflict. Alternatively, the precise timing of the initiation of the embryonic patterning process might be the driving force behind the use of a gamete-derived two-component system that initiates the patterning process by gamete fusion. Further experiments will shed light on the evolution of parental effects that influence embryonic patterning, axis formation, and suspensor development and thus shape the source–sink relationship of plants and their progeny during seed development.

**Perspectives**

- Parental factors strongly influence the apical-basal patterning of the Arabidopsis embryo.
- Maternal and paternal contributions control the ERECTA-YODA MAP kinase signaling pathway during zygote polarization, possibly highlighting a parental conflict during early seed development. Alternatively, parental factors may act as a two-component system to synchronize the patterning process with the fertilization event.
- Future research will show whether the parental effects on zygote polarization have evolved out of a need for optimized timing or are part of a parental conflict over nutrient allocation to the early embryo.

## Abbreviations

BSK: brassinosteroid signaling kinase
DAZ: Duo-Activated Zinc Finger
EPFL: epidermal patterning factor-like
HDG: homeodomain glabrous
MAP: mitogen-activated protein
MAPKK: MAP kinase kinase
RKD: RWP-RK Domain
SERK: somatic embryogenesis receptor kinase
TREE: Transcriptional Repressor of EIN3-dependent Ethylene-response
bHLH: basic helix-loop-helix
